# Staged Concept for Treatment of Severe Postsaphenectomy Wound Infection

**DOI:** 10.1155/2011/903839

**Published:** 2011-08-25

**Authors:** Thomas Schroeter, Sreekumar Subramanian, Michael A. Borger, Friedrich W. Mohr

**Affiliations:** Department of Cardiac Surgery, Heart Center Leipzig, University of Leipzig, 04289 Leipzig, Germany

## Abstract

The saphenous vein remains the most commonly used conduit in coronary artery bypass surgery. Vein harvest is a critical component with significant morbidity associated with leg wounds from open technique. Occurring complications are hematoma, postoperative pain, skin changes, neuropathy, and septic or nonseptic wound complications. Within the context of a recent case, we present our approach to postsaphenectomy wound management.

## 1. Introduction

Coronary artery disease (CAD) is one of the leading causes of morbidity and mortality in industrialized countries.Since the introduction of coronary artery bypass surgery (CABG) using reversed saphenous vein grafts by Favaloro [1], harvest techniques scarcely changed over the next 30 years. Only within the last 10 years has the application of minimally invasive and endoscopic techniques to reduce postoperative complications been gaining more attention [2]. However, due to concerns about the quality of the harvested vein, a significant proportion of saphenous vein is still being harvested in an open fashion, with septic and nonseptic wound complications occurring in 1–25%. These include hematoma, postoperative pain, skin changes, neuropathy, and cellulitis [3, 4]. Because CABG patients often suffer from additional risk factors including diabetes and adiposity, the management of wound complications is often challenging, which requires the implementation of a well-planned staged concept. Within the context of a recent case, we present our approach to postsaphenectomy wound management.

## 2. Case Report

**Figure 1:**
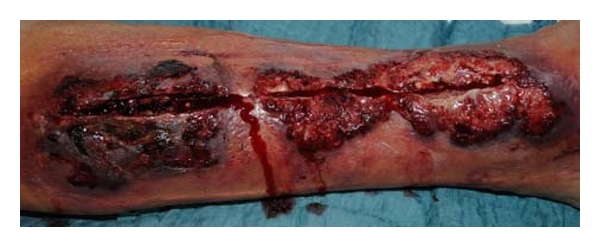
Post-saphenectomy wound complication before debridement: Infected hematoma and complete dehiscence of the cutaneous suture line.

A 68-year-old man with 2-vessel CAD was transferred emergently to our institution in the setting of an acute myocardial infarction (MI). His comorbidities included atrial fibrillation, thrombocytopathy, hyperthyroidism, and bilateral total knee replacement. He underwent emergent off-pump coronary artery bypass with left internal mammary artery (LIMA) and saphenous vein grafts (SVGs). Due to his preexisting platelet dysfunction, he required aggressive substitution of platelets and clotting factors. He underwent rethoracotomy on postoperative day number 2 for evacuation of a mediastinal hematoma. During the course of these events, he also developed an extensive saphenous vein site hematoma, which could be treated conservatively with pressure dressings. After adequate recovery, he was transferred to the normal wards, where he made good progress with mobilization. Unfortunately, he developed a wound complication in the region of the saphenectomy. This was not amenable to conservative therapy, so a graded approach to further treatment was employed (Figure 1).

## 3. Staged Concept

**Figure 2:**
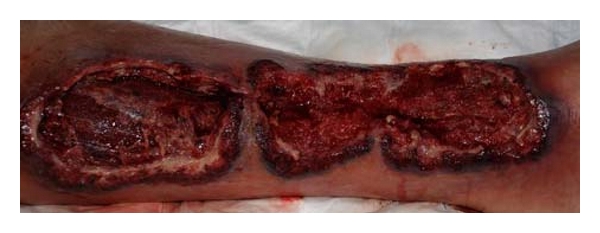
Wound appearance after extensive debridement, at the upper wound pole, the gastrocnemius muscle is exposed.

In the first stage, we obtained a specimen for microbiologic analysis and carried out an extensive debridement, which include removal of large patches of necrotic and infected skin on the medial leg (Figure 2). 

**Figure 3:**
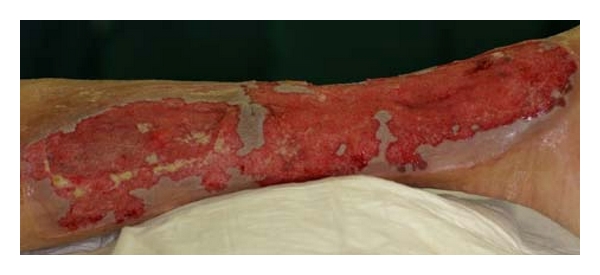
Appearance after 3 weeks of treatment with a vacuum dressing, intermittent suction of 75 mm Hg: Clean, well-granulated wound surfaces are evident, and the muscle is completely covered with granulation tissue.

Finally, a local disinfection with a mucosal disinfectant was carried out over 3 days. After obtaining a new sample for microbiologic analysis, a vacuum-assisted closure (VAC) with VacuSeal (KCI Medizinprodukte GmbH, Wiesbaden, Germany) and a maximal pressure of 75 mm Hg was implemented. The accompanying antibiotics were adjusted according to the first specimen culture results. In the first stage of treatment, we used a more porous type of foam since more cell detritus and necrotic tissue had to be removed. The dressing changes were performed every 3–5 days. After a large portion of the wound had developed granulation tissue, we carried out further VAC wound care with white foam to achieve further filling in of the defect and until wound sterility was achieved (Figure 3). 

Finally, wound coverage was achieved through 3 split-thickness skin grafts (STSGs), which were obtained from the thigh. This area had been covered during the initial healing phase by the vacuum dressing with minimal suction applied.

**Figure 4:**
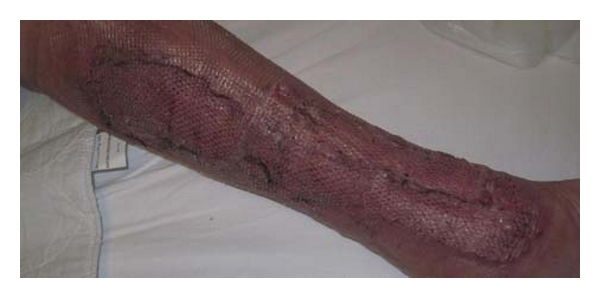
Wound appearance 7 days after split-thickness skin grafts and treatment with a light vacuum dressing to stabilize the skin grafts. A cosmetically acceptable result has been achieved, without functional limitations.

The patient was discharged in good condition, fully mobile, and with clean wounds and good incorporation of the STSG 5 weeks after the initial debridement (Figure 4).

## 4. Discussion

**Figure 5:**
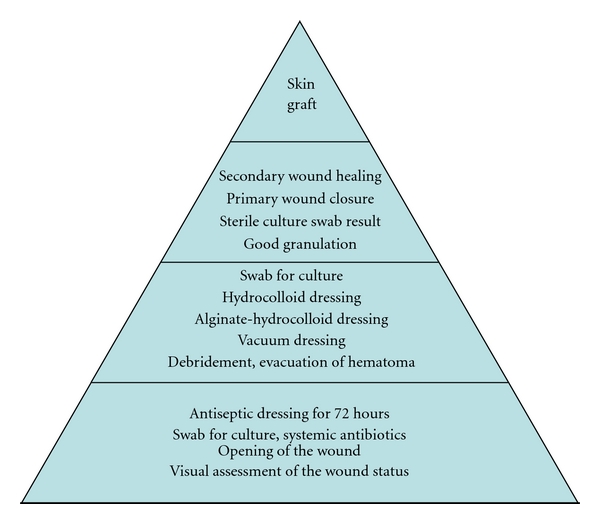
Staged schema for treatment of severe wound complications with escalation to skin graft coverage, depending on findings. With good progress of wound healing, a de-escalation of the therapy is possible at each level.

Postsaphenectomy wound complications may occur in 10–15% of patients. These consume significant additional medical and financial resources and result in a prolongation of the patient stay. In order to handle these wounds effectively, a standardized approach, which includes serial wound inspection, debridement, local antisepsis, granulation tissue ingrowth, and primary or secondary wound closure should be used (Figure 5). 

An important adjunct to debridement is the use of vacuum-assisted closure (VAC), regardless of whether the wound is superficial or deep, since that sets the foundation for an effective secondary closure [5]. In addition to the treatment of wound complications by a specially trained tream, the importance of prevention of wound complications has to be emphasized. The surgical procedure should be carefully selected, surgical harvest technique must disrupt surrounding tissues only slightly, wound closure has to be sufficiently layered, hematomas must be avoided and accompanying risk factors like obesity and diabetes must be appropriately managed. Judicious use of surgical drains, particularly in cases where a tissue flap has been inadvertently raised, and early evacuation of hematoma are important adjuncts that can prevent the development of pressure necrosis and subsequent catastrophic wound problems. The transition to minimally invasive vein harvest techniques (skin bridge or endoscopic harvest) has been rightly spurred by complications with open techniques and cosmetic concerns. This is appropriate as long as the vein quality with minimally invasive harvest remains suitable for CABG and that long-term patency is not compromised.

## 5. Conclusion

By implementing a staged approach catering to the various factors involved in wound complications, an adequate result may be obtained through the application of diverse resources even in wounds with extensive tissue damage and/or loss.

##  Disclosure

There are no competing interests for all authors. An ethical approval is not required, no patient related information will be provided, and all steps of the treatment are proven concepts and not part of a study.
